# Development and Validation of an Energy Consumption Model for Animal Houses Achieving Precision Livestock Farming

**DOI:** 10.3390/ani12192580

**Published:** 2022-09-27

**Authors:** Longhuan Du, Li Yang, Chaowu Yang, Chenming Hu, Chunlin Yu, Mohan Qiu, Siyang Liu, Shiliang Zhu, Xianlin Ye

**Affiliations:** 1 College of Architecture and Environment, Sichuan University, Chengdu 610065, China; 2 Sichuan Animal Science Academy, Chengdu 610065, China; 3 Sichuan Academy of Agricultural Sciences, Chengdu 610065, China

**Keywords:** energy consumption, indoor gas concentrations, environmental control, precision livestock farming

## Abstract

**Simple Summary:**

A customized thermal-dynamic model was developed in the present study based on the ISO13790 Standard to predict the energy consumption of poultry houses with indoor environment control. A validation test was performed in a layer house by applying sensors and meters to record the indoor environmental parameters including temperature, relative humidity, gas concentrations and energy consumption. The validation results indicated that the simulated environmental parameters agree well with the measured data showing a similar overall trend with limited discrepancies. Meanwhile, the difference in total energy consumption between the predicted and measured value was only about 10.6%, indicating the model was able to accurately estimate the energy demand during poultry farming. The proposed model enables farmers to quickly check and optimize their management strategies to achieve precision livestock farming from the energy consumption perspective.

**Abstract:**

Indoor environmental control is usually applied in poultry farming to ensure optimum growth conditions for birds. However, these control methods represent a considerable share of total energy consumption, and the trend of applying new equipment in the future for precision livestock farming would further increase energy demand, resulting in an increase in greenhouse gas emissions and management costs. Therefore, to ensure optimum efficiency of both energy use and livestock productivity, a customized hourly model was developed in the present study to interpret and analyze the electronically collected data. The modules for estimating indoor gas concentrations were incorporated into the present model, as this has not been properly considered in previous studies. A validation test was performed in a manure-belt layer house using sensors and meters to measure the indoor environmental parameters and energy consumption. The predicted results, including indoor temperature, relative humidity, carbon dioxide and ammonia concentrations, showed good agreement with the measured data, indicating a similar overall trend with acceptable discrepancies. Moreover, the corresponding differences between the measured and simulated energy consumption for heating, tunnel ventilation and base ventilation were 13.7, 7.5, and 0.1%, respectively. The total energy demand estimated by the model showed a limited discrepancy of approximately 10.6% compared with that measured in reality. Although human factors, including inspection, cleaning, vaccination, etc., were not included in the model, the validation results still suggested that the customized model was able to accurately predict the indoor environment and overall energy consumption during poultry farming. The validated model provides a tool for poultry producers to optimize production planning and management strategies, increase the production rate of unit energy consumption and achieve precision livestock farming from an energy consumption standpoint.

## 1. Introduction

The per capita annual meat consumption in China has rapidly increased in recent decades, and this trend is replicated across other East Asian countries, with regional per capita meat consumption expected to double over the next 20 years, according to the Global Food Policy Report [1]. The demand for animal proteins worldwide is expected to continue to rise in the upcoming decades due to the increasing global population [2,3]. To meet the mounting demand, intensive animal farming in livestock houses, where the indoor environment is controlled, is now widely applied in many countries. Compared with traditional farming, the main advantage of having livestock, such as broiler chickens, in a controlled environment is that the animals’ optimum growth conditions can be achieved with accuracy [4]. For example, in poultry farming, temperature control is crucial, especially for the first few weeks, since chicks are very sensitive to temperature variation. Broilers and layers suffer from hyperthermia or hypothermia when the indoor air temperature is higher or lower than the corresponding critical temperature, respectively, affecting bird production performance, physiology and welfare [5]. Moreover, indoor moisture strongly affects bird performance, and high values of relative humidity (RH) increase bird thermal stress, resulting in health problems and increased mortality [6]. Furthermore, the concentration of contaminants present in the poultry house, including harmful gases and airborne microorganisms, should also be kept below critical levels [7,8].

To guarantee a proper indoor climate, the air temperature, moisture and air quality must be controlled by ventilation, heating, cooling, etc. [9]. However, these control methods represent a considerable share of the total energy consumption of poultry farming. According to a recent study [10], environmental control in broiler houses accounts for 75.5% of the total electrical energy consumption, and this number is approximately 58.9% for laying hen farming. In addition, the corresponding proportions of electrical energy consumption resulting from environmental control in dairy cow farming and pig farming are 27.4% and 50.2%, respectively [10]. The energy demand in livestock farming is expected to further increase in the future due to the shift in technology and application of new equipment, such as electrostatic precipitators (ESPs) located at the air exhaust [11]. Therefore, the increase in energy consumption is no longer the only issue concerning the increase in animal production but is also related to the increase in greenhouse gas emissions [12], water consumption [13] and management costs [14]. Consequently, a comprehensive understanding and prediction of the energy consumption for intensive poultry farming with indoor climate control is essential from design and management standpoints to optimize the production process, optimize house design, save energy, improve overall efficiency and, finally, achieve sustainable development in the poultry industry.

Computer technology is an efficient method to provide fast insight into various problems instead of performing time-consuming and costly field measurements. Customized models and computational fluid dynamics (CFD) models have been widely applied in the poultry sector to investigate mortality rates [15], growth [16], indoor bioaerosol concentrations [17], airflow patterns [18], indoor temperatures and humidities [19], evaporative cooling systems [20], optimum ventilation strategies [21] and ammonia emissions [22]. Nevertheless, although many models have been built and applied to investigate various subjects, most of these studies provide limited information about how the examined topics affect overall energy consumption. Moreover, a dynamic model is required to take into account the sudden variation in the boundary conditions during the production cycle to predict the heat gains and losses through the thermal envelope of the poultry houses, which usually cannot be achieved by static models. More recently, a study [23] developed a thermodynamic model to investigate the humidity and mean indoor temperature for a single-story broiler barn, and their simulation results agree well with the experimental measurements. Another study [24] proposed an hourly model to predict the thermal behavior of a broiler house. The adopted time step of one hour was considered to be short enough to correctly take the variation in outdoor and indoor conditions into consideration, and the overall energy needed for heating and cooling was accurately estimated. In a study [25] applying Designer’s Simulation Toolkit (DeST) software to simulate the hourly heating and cooling load of a commercial layer hen house, DeST was demonstrated to be an effective and accurate tool for evaluating and predicting dynamic building loads for poultry houses. Unfortunately, DeST code was not publicly available. Based on the literature, a limited number of models are available for dynamically predicting energy consumption during poultry production. Moreover, to the best of the authors’ knowledge, the indoor gas concentrations have not been properly modeled or considered in the dynamic models available.

Therefore, the overall aim of this study was the following:

- Develop a customized model based on ISO 13790 (energy balance module, Section 2.5) to predict the energy consumption of poultry houses with indoor environmental control.
- Incorporate the modules for estimating the indoor ammonia and carbon dioxide concentrations into the model (Section 2.8). Incorporate the module for solar radiation into the model (Section 2.9). The related animal data were based on the performance of the local species and management strategies on the local requirements (Section 2.1, Section 2.2, Section 2.3, Section 2.4, Section 2.6.1 and Section 2.10). The ventilation module and moisture balance module were built according to Costantino et al.’s proposal [24] (Section 2.6.2, Section 2.6.3 and Section 2.7).
- Validate the model in terms of the indoor environmental parameters and overall energy consumption. Sixty days of continuous experimental measurements were conducted at a small-scale layer hen house to validate the model. Indoor and outdoor environmental parameters, including temperature, relative humidity, solar radiation, etc., were monitored and recorded together with the concentrations of ammonia and carbon dioxide. Additionally, energy consumption from ventilation fans, heaters and other equipment was also monitored.
- Provide a customized model for poultry producers to interpret the information captured, optimize management strategies and ensure optimum efficiency of both energy use and livestock productivity, achieving precision livestock farming from an energy consumption standpoint.

## 2. Materials and Methods

### 2.1. The Poultry House

**Figure 1:**
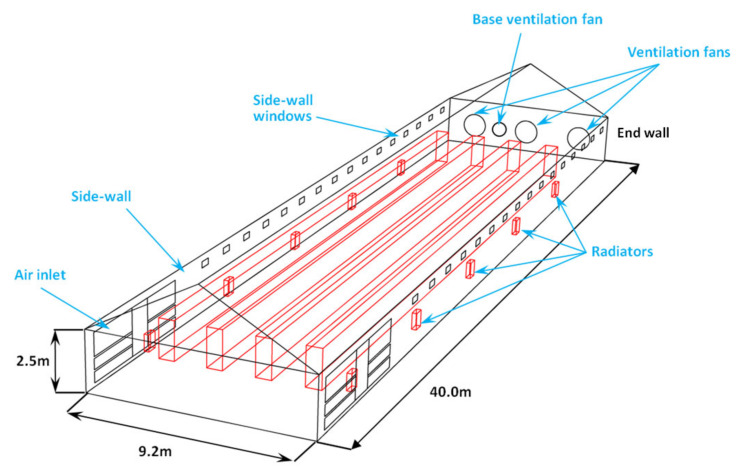
Schematic drawing of the modeled poultry house.

The modeled poultry house was an experimentally oriented manure-belt layer house located in Chengdu, Sichuan Province, China (30.47 N, 103.73 E). The dimensions of this east–west oriented house were length, L=40 m, width, W=9.2 m, height, and H=2.5 m (see Figure 1). Three tunnel ventilation fans, each with a diameter of 1.27 m (1200 W/fan,$\sim 42,000{\;m}^{3}/h\cdot \mathrm{fan}$), and a small base ventilation fan with a diameter of 0.6 m (350 W,$\sim 6000{\;m}^{3}/h\cdot \mathrm{fan}$) were installed at the end wall of the house for tunnel ventilation and base ventilation, respectively. Base ventilation was mainly employed to maintain temperature and relative humidity, and prevent the accumulation of harmful gases in winter. Tunnel ventilation is applied to control the indoor climate year round. Moreover, evaporative cooling pads (corrugated materials, in total $\sim 16{\;m}^{2}$) were installed at the air inlets located at the other side of the house, which were activated in hot weather and cooled the outdoor air that passed through by adiabatic saturation. With regard to indoor heating, 10 forced-fan radiators were used to carry out the heating task, as shown in Figure 1. The small fan installed at the back of each radiator was turned on to enable forced convection heat transfer between radiators and airflows when the indoor temperature dropped below the critical temperature and a heating load was needed. Furthermore, although the poultry house was equipped with 32 sidewall windows, they were not utilized in this study and kept closed during the validation test. In the house, there were 4 rows of animal-occupied zones, and each row had 3 tiers of cages raising up to 15,000 chicks of a local species (Dahen 699). The materials and thermal-physical characteristics of the house are provided in Table 1. Furthermore, the internal heat capacity of the poultry house was determined to be $40,500{\;\mathrm{KJ}\;K}^{-1}$, and default values were used for other parameters according to ISO 13790 [26].

### 2.2. Overall Model Structure

**Figure 2:**
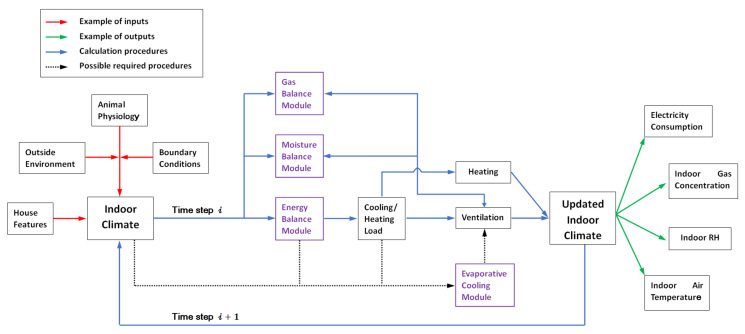
The flowchart of the customized model showing the key modules and simulation procedures.

The customized model developed in this study was based on the simple hourly method described in ISO 13790 [26]. The input data included the hourly outside environmental data (e.g., air temperature, air moisture, solar radiation intensity), building geometrical properties, thermal characteristics of the building, animal physiology (e.g., age, weight, heat production) and boundary conditions (e.g., set indoor temperature), which were determined from local management requirements, depending on the reared animal and expressed as a function of the animal age. For each time step of one hour, provided that the required boundary conditions, heat gain/loss and thermal behavior of the house envelope were known, the energy balance could be solved given the required heating or cooling load for indoor climate control. Moreover, at each time step, the moisture balance and gas balance (ammonia and carbon dioxide) were also simultaneously solved as long as the ventilation rate was determined. The indoor climate was updated at the end of the time step, and electricity consumption due to climate control was calculated considering the efficiency. A flow chart is provided in Figure 2 to show the overall simulation procedures with the examples of inputs and outputs at time step 𝒾. Detailed descriptions for each module and essential parts of the customized model are provided in the following sections.

### 2.3. Animal Body Weight and Heat Production

The reared birds were the parent stock of the local species characterized by partridge-like plumage and dark shanks. Under proper management and environmental conditions, the expected body weight of the birds is provided in Table 2. The customized model read the weight of the birds as input data as a function of their age (day), and a linear interpolation was applied for days within the week. Although heat production is generally better correlated with animal body area than body weight, body area changes frequently and is relatively hard to determine. Therefore, in this study, the customized model calculated the single animal total heat production $\emptyset_{s,\mathrm{tot}}$ as a function of the animal body weight $W_{b}$, and according to the study performed by Bartali et al. [27], $\emptyset_{s,\mathrm{tot}}$ for layer hens (in cage) was given by Equation (1) below.

(1) $$\emptyset_{s,\;\mathrm{tot}}=6.28\cdot W_{b}^{0.76}+25Y\;\left[W\right]$$

where Y is egg production, kg/bird·day. However, since the validation test was performed during the brooding stage (0~10 weeks) in the present study, the coefficient of egg production, Y, was zero. Equation (1) represents the total heat production of a single bird, including the latent and sensible heat emissions. Taking the number of birds, n, into consideration, which was the model input, the heat production of the flock could be easily calculated as $\emptyset_{f,\mathrm{tot}}=\emptyset_{s,\mathrm{tot}}\cdot n$ [W].

### 2.4. Indoor Temperature Management

**Figure 3:**
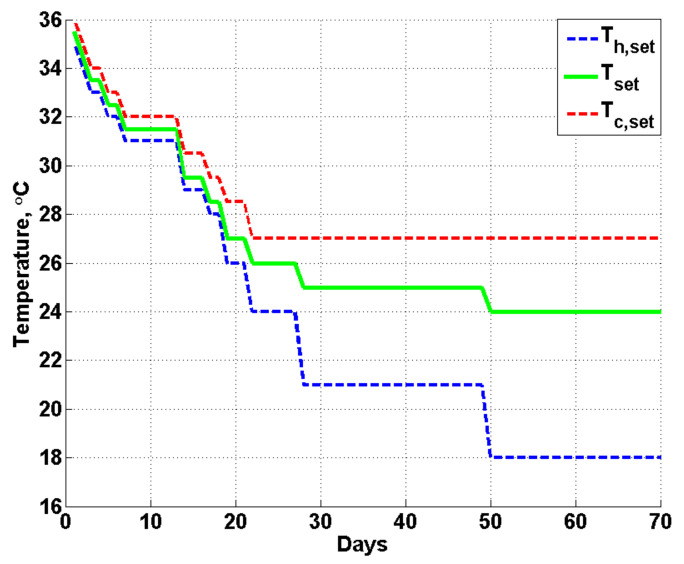
Indoor set temperature, $T_{\mathrm{set}}$, heating set temperature, $T_{h,\mathrm{set}}$ and cooling set temperature, $T_{c,\mathrm{set}}$.

Indoor climate control aims to provide reared animals with optimum environmental conditions to maximize performance. However, the required conditions are different at different stages of rearing. In this study, the indoor set temperature, $T_{\mathrm{set}}$, varied as a function of bird age, and a relatively higher temperature was required for the chicks at the beginning of the batch, while a relatively lower temperature was required when the birds grew up. A deadband in temperature was set wherein the temperature could fluctuate in free-running conditions. The lower value of the band was defined as the heating set temperature, $T_{h,\mathrm{set}}$, and upper value of the band was treated as the cooling set temperature, $T_{c,\mathrm{set}}$. Based on the local management experience for the local species, the indoor set temperature, $T_{\mathrm{set}}$, for optimum bird performance is illustrated in Figure 3 together with the $T_{h,\mathrm{set}}$ and $T_{c,\mathrm{set}}$ from 1 week of age to 10 weeks of age (70 days). As shown in Figure 3, at the beginning of the batch, the indoor temperature was set at approximately 35.5 °C, as the chicks are vulnerable to cold air. From week 8, the indoor temperature was lowered to approximately 24 °C, and this value was applied for the remaining time.

### 2.5. Energy Balance Module

**Figure 4:**
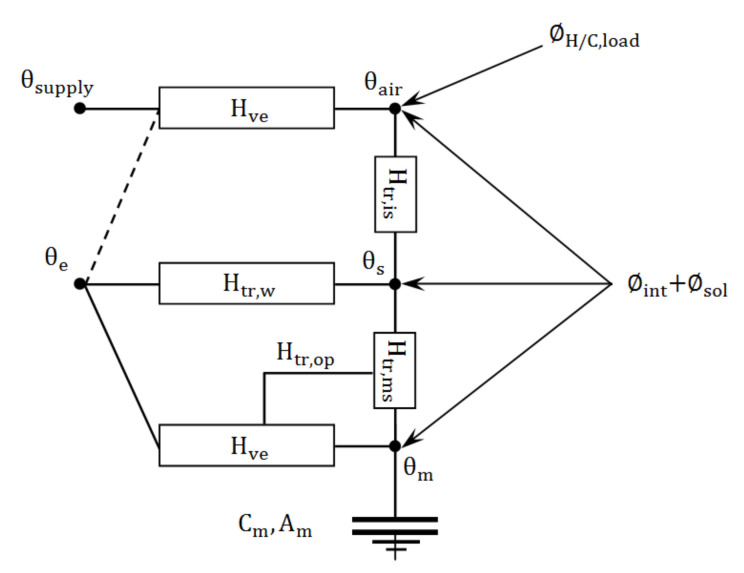
Schematic drawing of the 5R1C resistance-capacitance model showing the heat transfer between the internal and external environments.

In this study, the dynamic model for calculating the energy balance followed the simple hourly method described in ISO 13790 [26], which is based on similarity between the thermal behavior of the analyzed building and a resistance-capacitance (R-C) model. The heat transfer between the interior and exterior of the building could be calculated using a Crank–Nicholson scheme with a time step of one hour; the model makes a distinction between the internal air temperature and mean temperature of the internal surfaces, which enables its use in principle for thermal comfort verification and increases the accuracy by taking into account the radiative and convective parts of solar, lighting and internal heat gains. For each time step, the heating or cooling load ($\emptyset_{H/C,\mathrm{load}}$) was determined by calculating the need for heating or cooling power that needs to be supplied to, or extracted from, the building to maintain a certain minimum or maximum set-point temperature as described in Section 2.4. A schematic drawing of the heat transfer between the internal and external environments is illustrated in Figure 4.

As demonstrated in Figure 4, the heating or cooling load $\emptyset_{H/C,\mathrm{load}}$ is applied on the indoor air temperature node and represented as $\theta_{\mathrm{air}}$. The thermal losses by ventilation are calculated by the temperature difference between the supply air $\theta_{\mathrm{supply}}$ and indoor air $\theta_{\mathrm{air}}$ and as a function of the network resistance, $H_{\mathrm{ve}}$, which indicates the heat transfer coefficient by ventilation (it should be noted that the external air temperature, $\theta_{e}$, is equal to the supply air temperature, $\theta_{\mathrm{supply}}$, when evaporative cooling is not activated). Each node shown in Figure 4 represents a considered temperature, and the corresponding resistances, H, of the network indicate the heat transfer coefficients. Since the full set of equations for the simple hourly method was provided in ISO 13790 Annex C [26] and all aspects considered in the model or parameters shown in Figure 4 were described thoroughly in ISO 13790, readers should refer to ISO 13790 for detailed information.

### 2.6. Heating and Cooling

#### 2.6.1. Heating

**Figure 5:**
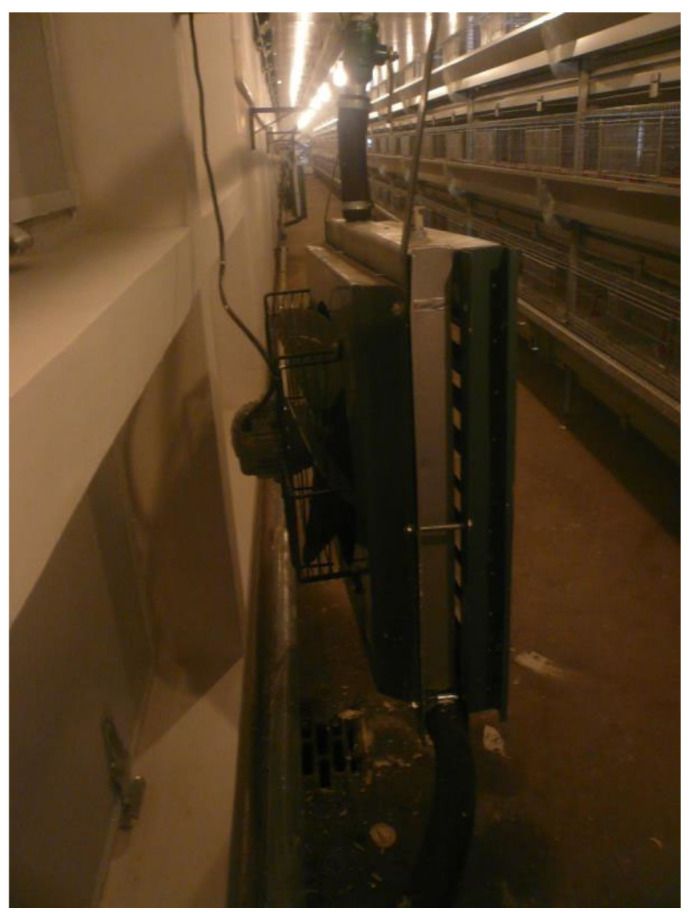
A small fan was installed at the back of the radiator.

Heating was usually required during the first few weeks, and the heating task was performed by the 10 radiators (Lucheng Technology, SF-100, Jinan, China), as shown in Figure 1. In the early days of the batch, the small fan (100 W/fan) installed at the back of each radiator (see Figure 5) would be automatically turned on to enable the forced convection heat transfer as long as the indoor air temperature $\theta_{\mathrm{air}}$ dropped below the critical temperature, $T_{h,\mathrm{set}}$, as shown in Figure 3. Under this circumstance, the energy consumption, $\emptyset_{H,\mathrm{load}}$, from the radiators, or more specifically from the electric boiler (Lucheng Technology, KTP-2, Jinan, China), and the small fans ($\emptyset_{\mathrm{samll}\;\mathrm{fans}}$) were both taken into account during the modeling. Furthermore, during the first few weeks, the radiators would be treated as indoor heat sources since the circulation water temperature was kept at a certain value by the boiler at all times. Therefore, this additional energy consumption by the boiler was expressed in Equation (2) as

(2) $$E_{\mathrm{natural}}=\overset{n}{\underset{i=1}{\sum }}{\left[\emptyset_{\mathrm{natural}}\cdot 1h\right]}_{i}=\overset{n}{\underset{i=1}{\sum }}{\left[H\cdot A_{r}\cdot \Delta T\cdot 10^{-3}\cdot 1h\right]}_{i}\;\left[\mathrm{kWh}\right]$$

where i is a certain time step, 1 h was used as the time step in the model; $\emptyset_{\mathrm{natural}}$ is the heat transfer due to natural convection for a certain time step, H is the coefficient for natural convection heat transfer, $A_{r}$ is the total surface area of the radiator and ΔT is the temperature difference between the air and the radiator surface. After the 4th week, the radiators and boiler were totally turned off.

Finally, the total heating energy, $E_{\mathrm{heating}}$, could be estimated by summing up the heating load ($\emptyset_{H,\mathrm{load}}+\emptyset_{\mathrm{samll}\;\mathrm{fans}}$) required at each time step of the analyzed period and the energy consumed by the boiler to keep the circulating water at a certain temperature:

(3) $$E_{\mathrm{heating}}=\sum_{j=1}^{n}{\left[\left(\emptyset_{H,\mathrm{load}}+\emptyset_{\mathrm{samll}\;\mathrm{fans}}\right)\times 1h\right]}_{j}+\sum_{i=1}^{n}{\left[\emptyset_{\mathrm{natural}}\times 1h\right]}_{i}\left[\mathrm{kWh}\right]$$

where i, j is a certain time step, and 1 h was the time step used in the model.

#### 2.6.2. Base Ventilation

In this study, the base ventilation fan was activated all the time during the validation test and in the model, resulting in a constant ventilation rate of $Q_{\mathrm{base}}$ and a constant power requirement.

#### 2.6.3. Tunnel Ventilation

The cooling load,$\;\emptyset_{C,\mathrm{load}}$, calculated by the model at a certain time step was simply a theoretical value and used to determine the ventilation flow rate. In this study, the cooling task was performed by three tunnel ventilation fans installed at the end wall of the poultry house (see Figure 1), and they were activated when all the following conditions were met:

- the indoor air temperature was higher than the critical temperature ($\theta_{\mathrm{air}}>T_{c,\mathrm{set}}$)
- the cooling load was larger than zero ($\emptyset_{C,\mathrm{load}}>0$)
- the outdoor air temperature, $\theta_{e}$, was sufficiently lower than the critical temperature, $T_{c,\mathrm{set}}$, (${\mathrm{it}\;\mathrm{should}\;\mathrm{be}\;\mathrm{noted}\;\mathrm{that}\;\theta }_{\mathrm{supply}}=\theta_{e}$ when evaporative cooling was not activated).

With regard to the last condition, the term ‘sufficiently lower’ meant that $T_{c,\mathrm{set}}-\theta_{e}\geq 1\;{}^{\circ}C$ in this study. An extremely small difference between $\theta_{e}$ and $T_{c,\mathrm{set}}$ would result in an infinite value of the ventilation flow rate, which would make ventilation unfeasible. Moreover, it should be noted that in reality, there was a maximum ventilation flow rate that could be generated by the three ventilation fans; therefore, an upper limit was also set accordingly in the customized model. When all above conditions were met and verified, the ventilation flow rate at this time step was then determined as:

(4) $$Q_{\mathrm{tunnel}}=\frac{\left|\emptyset_{C,\mathrm{load}}\right|}{c_{\mathrm{air}}\cdot \left(T_{c,\mathrm{set}}-\theta_{e}\right)\cdot \rho_{\mathrm{air}}}m^{3}{\;s}^{-1}$$

where $c_{\mathrm{air}}$ is the air-specific heat capacity (J/kg·°C) and $\rho_{\mathrm{air}}$ is the air density ($\mathrm{kg}/m^{3}$).

#### 2.6.4. Evaporative Cooling

In hot summers, the outdoor air temperature might rise above $T_{c,\mathrm{set}}$, resulting in the cooling task not being fulfilled by tunnel ventilation alone. Under this circumstance, evaporative cooling should be activated to reduce the supply air temperature and further cool the enclosure. Thus, evaporative cooling was activated when the following conditions were met:

- $\theta_{\mathrm{air}}>T_{c,\mathrm{set}}$
- $\emptyset_{C,\mathrm{load}}>0$
- $T_{c,\mathrm{set}}-\theta_{e}\geq 1.0\;{}^{\circ}C$ was not satisfied or $T_{c,\mathrm{set}}-\theta_{e}<1.0\;{}^{\circ}C$

Evaporative cooling pads cooled the outdoor airflow that passes through them by adiabatic saturation. The supply air temperature, $\theta_{\mathrm{supply}}$, after the evaporative cooling pads was determined as a function of the outdoor dry-bulb temperature,${\;\theta }_{e,\mathrm{db}}$, the outdoor wet-bulb temperature, $\theta_{e,\mathrm{wb}}$, and the direct saturation effectiveness, ε, of the cooling pads, which was expressed in Equation (5) as

(5) $$\varepsilon =\frac{\theta_{e,\mathrm{db}}-\theta_{\mathrm{supply},\mathrm{db}}}{\theta_{e,\mathrm{db}}-\theta_{e,\mathrm{wb}}}\cdot 100\%\left[-\right]$$

Generally, ε is affected by the velocity of the air passing through the pads, the thickness of the pad and dust on the surface (maintenance). In this study, ε values from the datasheet provided by the manufacturer were directly used in the model, and the supply air temperature after the cooling pads could be calculated as

(6) $$\theta_{\mathrm{supply},\mathrm{db}}=\theta_{e,\mathrm{db}}-\varepsilon \cdot (\theta_{e,\mathrm{db}}-\theta_{e,\mathrm{wb}})\;[{}^{\circ}C]$$

Finally, under this circumstance, the ventilation flow rate was expressed in Equation (7):

(7) $$Q_{\mathrm{ec}}=\frac{\left|\emptyset_{C,\mathrm{load}}\right|}{c_{\mathrm{air}}\cdot \left(T_{c,\mathrm{set}}-\theta_{\mathrm{supply}}\right)\cdot \rho_{\mathrm{air}}}\;[m^{3}{\;s}^{-1}]$$

In conclusion, under different boundary conditions, the indoor effective ventilation flow rate, $Q_{\mathrm{eff}}$, was different and summarized as

- $Q_{\mathrm{eff}}=Q_{\mathrm{base}}$ (cooling load is not required)
- $Q_{\mathrm{eff}}=Q_{\mathrm{base}}+Q_{\mathrm{tunnel}}$ (tunnel ventilation is required only)
- $Q_{\mathrm{eff}}=Q_{\mathrm{base}}+Q_{\mathrm{ec}}$ (evaporative cooling is activated)

### 2.7. Moisture Balance Module

The total heat production from a bird, $Q_{s,\mathrm{tot}}$, can be further divided into sensible, $Q_{s,\mathrm{sen}}$, and latent, $Q_{s,\mathrm{lat}}$, heat dissipation. $Q_{s,\mathrm{sen}}$ was proportional to the temperature difference between the animal surface and ambient air, indicating that $Q_{s,\mathrm{sen}}$ would become zero if the surrounding air temperature was equal to the animal surface temperature. Furthermore, $Q_{s,\mathrm{lat}}$ dissipated in the form of moisture, and $Q_{s,\mathrm{lat}}$ increased with increasing ambient temperature to maintain animal heat balance and body temperature. To calculate the moisture dissipation of the birds in the poultry house, the sensible fraction of the heat emission, R, was determined according to the study performed by Bartali et al. [27] as

(8) $$R=f_{1}\cdot T_{\mathrm{set}}^{5}+f_{2}\cdot T_{\mathrm{set}}^{4}+f_{3}\cdot T_{\mathrm{set}}^{3}+f_{4}\cdot T_{\mathrm{set}}^{2}+f_{5}\cdot T_{\mathrm{set}}^{1}+f_{6}$$

where coefficients $f_{1}\sim f_{6}$ were determined from the polynomial fit curve, and the values are reported in Table 3.

Once the sensible fraction, R, was known, sensible and latent heat emissions for a single bird could be easily determined by Equation (9) and Equation (10), respectively. Furthermore, the vapor mass production rate of the whole flock, $m_{f,\mathrm{vapor}}$, could then be calculated using Equation (11):

(9) $$\emptyset_{s,\mathrm{sen}}=R\cdot \emptyset_{s,\mathrm{tot}}\;\left[W\right]$$

(10) $$\emptyset_{s,\mathrm{lat}}=\emptyset_{s,\mathrm{tot}}-\emptyset_{s,\mathrm{sen}}\;\left[W\right]$$

(11) $$m_{f,\mathrm{vapor}}=\frac{\emptyset_{s,\mathrm{lat}}\cdot n\cdot 10^{-3}}{h_{\mathrm{vapor}}}\;[{\mathrm{kg}\;s}^{-1}]$$

where $h_{\mathrm{vapor}}$ is the specific enthalpy of the water vapor (kJ/kg) at the set air temperature $T_{c,\mathrm{set}}$, which is the input data for the model. At each time step, the model solved the humidity mass balance by taking the humidity ratio of the inlet air, $x_{\mathrm{in}}$, vapour production, $m_{f,\mathrm{vapor}}$, and the humidity ratio of the outlet air, $x_{\mathrm{out}}$, into consideration. The humidity ratio could be determined by Equation (12), and overall moisture balance in the poultry house was then expressed in Equation (13) as

(12) $$x=\frac{0.62198\cdot P_{w}}{P_{a}-P_{w}}$$

(13) $$Q_{\mathrm{eff}}\cdot \rho_{\mathrm{air}}\cdot x_{\mathrm{in}}+m_{f,\mathrm{vapor}}=Q_{\mathrm{eff}}\cdot \rho_{\mathrm{air}}\cdot x_{\mathrm{out}}\;[{\mathrm{kg}\;s}^{-1}]$$

where $P_{w}$ is the partial pressure of water vapor in moist air, Pa, $P_{a}$ is the atmospheric pressure of moist air, Pa, and $Q_{\mathrm{eff}}$ is the effective ventilation rate as introduced in Section 2.6 at the current time step, $m^{3}/s$.

### 2.8. Gas Balance Module

The ammonia (${\mathrm{NH}}_{3}$) and carbon dioxide (${\mathrm{CO}}_{2}$) emissions inside the layer hen house could cause bird and worker exposure to levels that exceed indoor air quality thresholds, resulting in related diseases and reduced animal performance. It is important to understand and predict the gas emissions and concentrations during the whole batch under various environmental conditions for the effective management of harmful gases and establishment of fair regulations. Therefore, modules were built in this customized model to simulate the dynamic change in the concentrations of carbon dioxide and ammonia inside the poultry house using recently established methods.

#### 2.8.1. Ammonia

The prediction of ammonia emissions inside the house was based on the mechanistic model that was especially developed for layer hen houses by Tong et al. [28]. A detailed description of each aspect that should be considered when simulating ammonia emissions is not presented here, but readers can refer to reference [28] for more information. The final overall equation for estimating the ammonia emission rate (ER) from layer manure was expressed in Equation (14) as

(14) $$\mathrm{ER}=\left\{{\left[1+\frac{10^{-\mathrm{pH}}}{\alpha \cdot K_{d0}}\right]}^{-1}\times \left(\frac{\mathrm{TAN}}{1-\frac{\mathrm{MC}}{100}}\right)\times K_{h}\times 1000\times \left(\frac{100-\mathrm{MC}}{\mathrm{MC}}\right)-C_{g,\infty }\right\}\times f_{K_{G}}\;\left[{\mathrm{mg}\;m}^{-2}h^{-1}\right]$$

where pH is the manure pH; MC is the moisture content of the manure, %; α is the dissociation constant ratio calculated as a function of MC and pH; $K_{d0}$ is the dissociation constant of the $H_{2}O-{\mathrm{NH}}_{3}$ solution; TAN is the wet-based total ammonia nitrogen, having a constant value of 6943 μg/g as suggested by Tong et al. [28]; $K_{h}$ is Henry’s law constant; $C_{g,\infty }$ is the outdoor ammonia concentration, $\mathrm{mg}/m^{3}$; and $f_{K_{G}}$ is the convective mass transfer coefficient determined as a function of air temperature and air velocity, m/h. Once the ER value was determined, the mass ($M_{{\mathrm{NH}}_{3}}$) and concentration ($V_{{\mathrm{NH}}_{3}}$) of ammonia at each time step were expressed by Equation (15) and Equation (16), respectively:

(15) $$M_{{\mathrm{NH}}_{3}}=\mathrm{ER}\cdot A_{\mathrm{manure}}\;[{\mathrm{mg}\;h}^{-1}]$$

(16) $$V_{{\mathrm{NH}}_{3},\mathrm{out}}=[\left(\frac{M_{{\mathrm{NH}}_{3}}}{\rho_{\mathrm{ammonia}}})/(Q_{\mathrm{eff}}*3600\right)\;]+{\;V}_{{\mathrm{NH}}_{3},\mathrm{in}}\;\left[\mathrm{ppm}\right]$$

where $A_{\mathrm{manure}}$ is the total area of manure on the manure belt at the time step of$\;𝒾,{\;m}^{2}.{\;V}_{{\mathrm{NH}}_{3},\mathrm{in}}$, $V_{{\mathrm{NH}}_{3},\mathrm{out}}$ is the ammonia concentration at the inlet (model input data) and outlet, ppm, respectively, and $\rho_{\mathrm{ammonia}}$ is the density of ammonia, mg/mL. As seen in Equations (15) and (16), to accurately predict or simulate ammonia emissions, the value of $A_{\mathrm{manure}}$ at each time step was essential and should be scientifically determined. According to a recent study performed by Yang et al. [29], the manure coverage proportion (MCP) on the manure belt for typical layer houses within 48 h could be estimated by Equation (17):

(17) $${\mathrm{MCP}}_{48}=P_{1}\times h^{4}+P_{2}\times h^{3}+P_{3}\times h^{2}+P_{4}\times h+P_{5}$$

where h is the time (hours) after the most recent manure removal; the values of the coefficients of${\;P}_{1}\sim P_{5}$ are provided in Table 4. For the validation test in this study, the manure belt was also cleared every 48 h, and the total manure belt area was approximately 340${\;m}^{2}$; therefore, the manure area ($A_{\mathrm{manure}}$) in the layer house at time step 𝒾 was calculated to be

(18) $$A_{\mathrm{manure}}={\mathrm{MCP}}_{𝒾}\times 340\;\left[m^{2}\right]$$

#### 2.8.2. Carbon Dioxide

The estimation of carbon dioxide (${\mathrm{CO}}_{2}$) emissions in the poultry house was based on the principle of indirect animal calorimetry as detailed in reference [30]. This method assumes that the metabolic heat production of nonruminants is related to the oxygen ($O_{2}$) consumption and ${\mathrm{CO}}_{2}$ production by the animal. Moreover, the respiratory quotient (RQ), which is the ratio of ${\mathrm{CO}}_{2}$ production to $O_{2}$ consumption, is defined in Equation (19) as

(19) $$\mathrm{RQ}=\frac{{\mathrm{CO}}_{2}}{O_{2}}\;\left[-\right]$$

**Figure 6:**
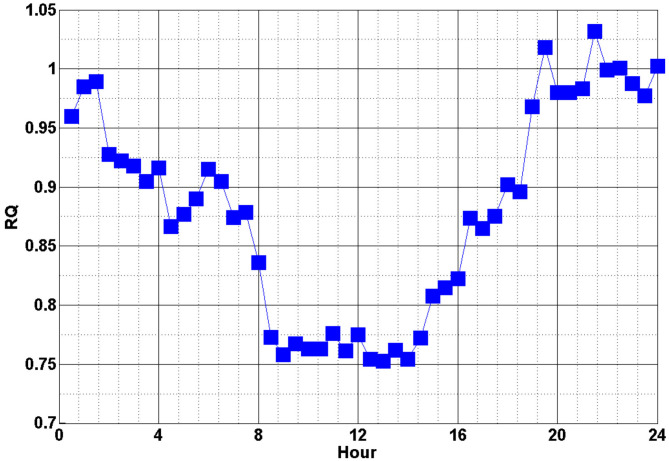
The applied RQ value in a day [32].

The ${\mathrm{CO}}_{2}$ concentration at the building outlet was then expressed in the following equations:

(20) $$\overset{\cdot }{V_{{\mathrm{CO}}_{2}}}=\frac{\emptyset_{f,\mathrm{tot}}}{\frac{16.18}{\mathrm{RQ}}+5.02}\;[{\mathrm{mL}\;s}^{-1}]$$

(21) $$V_{{\mathrm{CO}}_{2},\mathrm{out}}=\overset{\cdot }{(V_{{\mathrm{CO}}_{2}}}/Q_{\mathrm{eff}})+V_{{\mathrm{CO}}_{2},\mathrm{in}}\;\left[\mathrm{ppm}\right]$$

where $V_{{\mathrm{CO}}_{2},\mathrm{in}}$ is the ${\mathrm{CO}}_{2}$ concentration at the inlet, which was experimentally measured (model input data), ppm. The value of RQ varies theoretically from 0.71 to 1.3 depending on the metabolic rate, feed intake and individual status of the animal [31]. In this study, the applied RQ value during a day (24 h) was based on the study performed by Xin et al. [32], as shown in Figure 6. Higher RQ values were measured at night than during the daytime, which is probably associated with the greater energy retention and thus tissue deposition during this period [32].

### 2.9. Solar Radiation

The solar heat gains through opaque building elements, $\emptyset_{\mathrm{sol}}$, should not be underestimated for summer cooling or summer thermal comfort calculations, especially for poultry houses that are built with non-insulating material or traditional material. For the completeness of the model, these additional heat gains were carefully modeled in this study and applied in the energy balance module, as shown in Section 2.5 and Figure 4. The heat flow by solar gains through the poultry house was calculated by Equation (22) as [26]:

(22) $$\emptyset_{\mathrm{sol}}=A_{\mathrm{sol}}\cdot F_{\mathrm{sh}}\cdot I_{\mathrm{sol}}-F_{r}\cdot \emptyset_{r}\;\left[W\right]$$

where $A_{\mathrm{sol}}$ is the effective collecting area of the building, $m^{2}$, which is linearly related to the projected area of the building wall ($A_{c}$) perpendicular to the sunlight and other physical parameters of the wall material, as detailed in ISO 13790 [26]. $I_{\mathrm{sol}}$ is the solar irradiance, which was experimentally measured in this study during the whole batch period, $W/m^{2}$. $F_{\mathrm{sh}}$ is the shading reduction factor, and $F_{r}\cdot \emptyset_{r}$ is the heat flow due to the thermal radiation to the sky from the building, which is clearly explained in ISO 13790 ([26] Section 11.3.2) and will not be detailed here. Since $A_{\mathrm{sol}}$, or more specifically $A_{c}$, varies with the solar trajectory, it was important to take the position of the sun and dynamic value of $A_{c}$ into consideration when modeling the energy consumption of the poultry house for a relatively long time.

**Figure 7:**
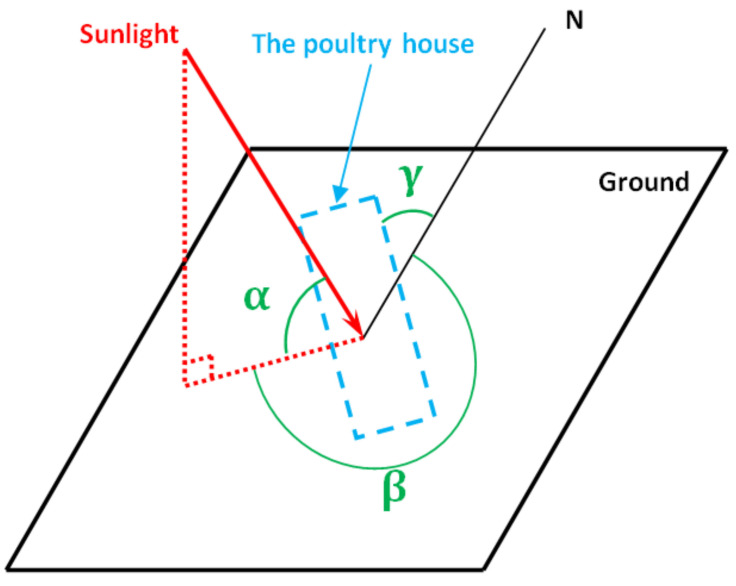
Schematic drawing of the solar elevation angle, α, and solar azimuth angle, β.

As shown in Figure 7, to calculate the real-time value of $A_{c}$, the current solar elevation angle, α, and solar azimuth angle, β, must be determined. According to reference [33], the solar elevation angle, α, which is the angular height of the sun in the sky measured from the horizon, can be calculated by using Equation (23):

(23) $$\sin\alpha =\sin\varphi \sin\delta +\cos\varphi \cos\delta \cos\omega \;\left[-\right]$$

where φ is the latitude of the building, $\omega ={\left[15\cdot \left(t-12\right)\right]}^{{}^{\circ}}$, and t is the current time, $t\in \left[0,\;24\right)$. δ is the declination angle (varies seasonally due to the tilt of the Earth on its axis of rotation and the rotation of the Earth around the sun), which can be determined by Equation (24):

(24) $$\delta =23.45\cdot \sin\left(\frac{2\pi \left(284+n\right)}{365}\right)\left[{}^{\circ}\right]$$

where n is the date number counting from 1 January, $n\in \left[1,365\right]$. Knowing the solar elevation angle, α, the solar azimuth angle, β (the compass direction from which the sunlight was coming) can be calculated by Equations (25) and (26):

(25) $$\beta =\mathrm{arccosB}\;\left[{}^{\circ}\right]\;\;\;\;\;\omega <0$$

(26) $$=360^{{}^{\circ}}-\mathrm{arccosB}\;\left[{}^{\circ}\right]\;\;\;\omega \geq 0$$

where B is expressed in Equation (27):

(27) $$B=\frac{\sin\delta -\sin\alpha \sin\varphi }{\cos\alpha \cos\varphi }\;\left[{}^{\circ}\right]$$

Finally, the dynamic $A_{c}$ can be determined as

(28) $$A_{c}=H\cdot L\cdot \sin\left(360-\beta -\gamma \right)\;[m^{2}]$$

where H and L are the height and length of the building, respectively, and γ is the angle between the building orientation and the north direction, as shown in Figure 7.

### 2.10. System Performance

To compare the model’s estimated heating energy consumption, $E_{\mathrm{heating}}$, with that measured in reality, a conversion coefficient ($\mu_{\mathrm{boiler}}=\frac{1}{\mathrm{efficency}\;\mathrm{of}\;\mathrm{electrical}\;\mathrm{energy}\;\mathrm{to}\;\mathrm{thermal}\;\mathrm{energy}\;\mathrm{of}\;\mathrm{water}\;}$) was applied for the boiler, and a transmission coefficient, $\mu_{\mathrm{pipe}}$, was also applied for the circulation piping system considering the heat loss. Finally, the final heating energy consumption was expressed in Equation (29) as

(29) $$E_{\mathrm{heating},\;\mathrm{final}}={\;E}_{\mathrm{heating}}\cdot \mu_{\mathrm{boiler}}\cdot \mu_{\mathrm{pipe}}\;\left[\mathrm{kWh}\right]$$

where $\mu_{\mathrm{boiler}}$ was equal to $\frac{1}{0.94}=1.06$ according to the manufacturer of the boiler, and $\mu_{\mathrm{pipe}}$ was assumed to be $\frac{1}{0.95}=1.05$ considering that the pipes were well insulated. With regard to the cooling energy consumption, once the ventilation fans were activated, the electrical energy was consumed based on the rated power of the fans, and the total cooling energy consumption could be calculated as the sum of the energy consumption of the fans for certain time steps of the analyzed period. It should be noted that the energy consumption for the evaporative cooling system was neglected in this study, as it only works under limited circumstances and responsible for a small fraction of the total energy consumption. A summary is provided in Table 5 to illustrate the modeled aspects for calculating the final system energy consumption.

## 3. Case Study—Validation Test

The validation test was performed in the poultry house shown in Figure 1. The whole house was preheated to approximately 37 °C before the 14,000 chicks were moved in. The test started from 1 week of age to approximately 9 weeks of age (60 days). Both environmental and energy data were recorded, and the considered period was believed to be adequate for model validation since all equipment for climate control (including all fans, radiators and evaporative pads) was used and different outdoor conditions were registered.

**Figure 8:**
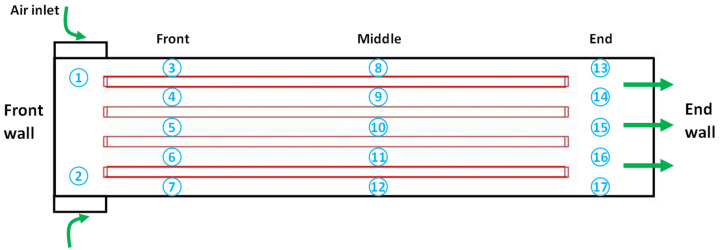
Schematic drawing of the locations of the sensors inside the analyzed poultry house. No. 1~No. 17 indicate the locations of the sensors.

The indoor environmental data, including gas concentrations, were collected by sensors installed at various locations in the house, as shown in Figure 8. The indoor air temperature and relative humidity (RH) were monitored at the air inlets positioned in the front, middle and end of the house (location No.1 ~ No.17) to provide a detailed overall profile. For poultry houses with tunnel ventilation, the indoor gas concentrations would reach the maximum values at the end of the house; therefore, the concentrations of ${\mathrm{NH}}_{3}$ and ${\mathrm{CO}}_{2}$ were only monitored at the air inlet and end of the house (No.1, No.2 and No.13 ~ No.17). The maximum value recorded at the back end of the house represented the indoor gas concentrations, while the values measured at the inlet were used as references. The outdoor environmental data were collected by a small-scale portable meteorological station, and the solar radiation intensity was monitored by a solar radiometer. All of the above monitored data were transmitted to a central processing/recording system, and the historical data could be reviewed at any time during/after the test. Detailed information about the environmental data collection is summarized in Table 6. The environmental data were collected at a frequency of $0.06\;\mathrm{Hz}\;\left(\mathrm{every}\;1\;\min\right)$, which is believed to be sufficient for validation of the hourly model.

In terms of energy consumption for the climate control of the poultry house, which was mainly in the form of electricity, several electricity meters were applied in the electrical lines. The instantaneous data were transmitted to the central processing/recording system, and the cumulative electrical energy consumption could be calculated and reviewed. Detailed information about energy monitoring is provided in Table 7. Moreover, other essential data required for the model input or model validation are summarized in Table 8.

## 4. Results and Discussion

**Figure 9:**
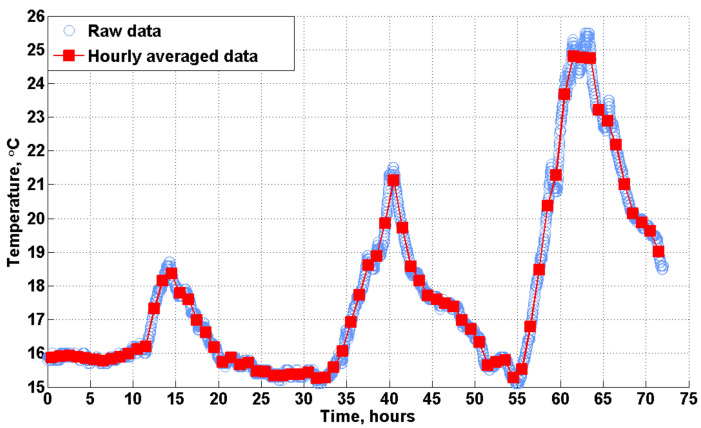
Examples of the hourly averaged data.

To provide a clear and direct comparison with those data predicted by the customized hourly model, the raw data measured and recorded every minute by the sensors were also averaged every hour, as shown in the example in Figure 9. This format of hourly averaged data is applied in the results.

### 4.1. Indoor Temperature (T) and Relative Humidity (RH)

**Figure 10:**
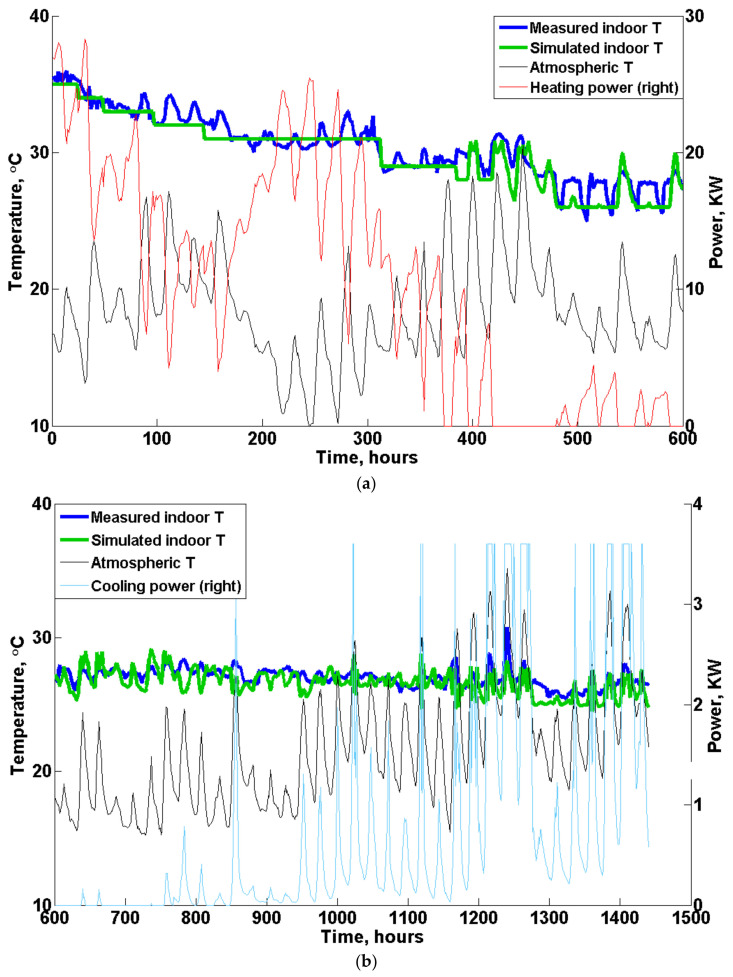
Temperature validation results. (**a**) 0~600 h, (**b**) 600~1440 h. Note: The base ventilation is not included in the figure. The maximum rated power of tunnel ventilation was 3.6 kW.

The final results of the measured and simulated indoor temperature (T) during the validation test are illustrated in Figure 10a for 0~600 h and in Figure 10b for 600~1440 h. Since the required indoor temperature, $T_{\mathrm{set}}$, for the first three weeks was approximately 30~35 °C, which is much higher than the outdoor temperature (or atmospheric temperature), the value of the heating set temperature ($T_{h,\mathrm{set}}$), as shown in Figure 3, was used in the customized model to calculate the hourly minimum required heating power, ensuring that the indoor temperature met the rearing condition. As a purely theoretical calculation in the model, no more heat would be added into the poultry house when the temperature reached $T_{h,\mathrm{set}}$, and constant indoor temperature values were predicted at the beginning of the batch, as shown in Figure 10a. In contrast, the temperature measured in reality fluctuated around $T_{\mathrm{set}}$ due to the hysteresis effect resulting from heating or cooling. In addition, although the indoor temperature simulated by the model indicated contact values for the first three weeks, the predicted heating power varied with the atmospheric temperature, demonstrating an inverse relationship, as can be clearly seen by the red and black lines in Figure 10a. Furthermore, with an increase in heat production from the birds and in outside temperature, the required heating power decreased significantly after approximately 400 h, and no more heat was required after 600 h.

During hours 600~1440 of the validation test, the temperature deadband became larger, as shown in Figure 3, and the indoor temperature could fluctuate in free-running conditions in the deadband region. For a period of approximately 600~800 h, the atmospheric temperature played a major role in affecting the indoor temperature, as demonstrated both by the measurements and model simulation, as shown in Figure 10b. No heating and only slight cooling power (except the base ventilation) was required during this period, and the predicted temperature matched well with the measured data, showing limited discrepancies. Moreover, with the increase in the birds’ body weight (heat production) and outdoor temperature, the required cooling power increased considerably after approximately 1000 h. For certain periods at the end of the test, as demonstrated in Figure 10b, the cooling power of the tunnel ventilation reached the maximum value or rated power of 3.6 kWh, and the evaporative cooling system would be activated if the indoor temperature could still not be controlled by ventilation alone.

**Figure 11:**
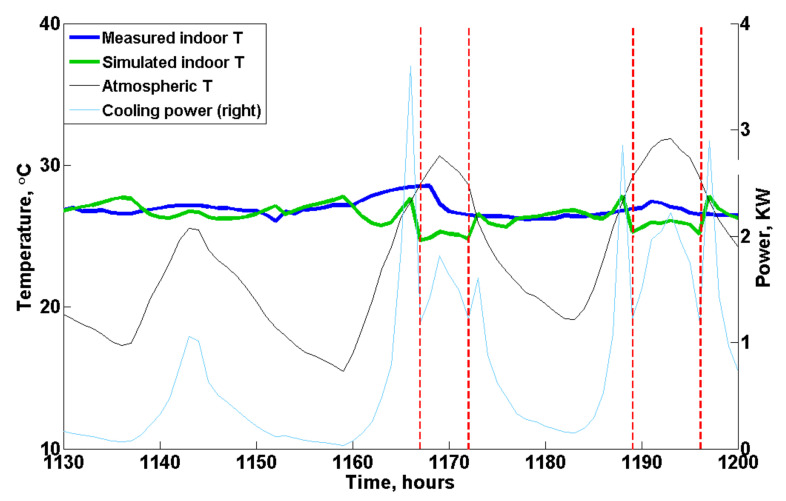
An example of the effect of evaporative cooling on the indoor temperature. The red dashed line indicates the activation of evaporative cooling in the customized model. Note: The base ventilation is not included in the figure.

An example of the effect of evaporative cooling on the indoor temperature is shown in Figure 11. A sudden decrease in the indoor temperature was simulated by the model at 1167 h (indicated by the red dashed line in Figure 11), while the atmospheric temperature kept rising in the following hours, which indicated that the supply air temperature in the poultry house decreased significantly resulting from the activation of evaporative cooling. Furthermore, the temperature measured by the sensors also indicated that evaporative cooling was activated in the poultry house in reality but approximately 1 h later at 1168 h, as shown in Figure 11. Moreover, when evaporative cooling was activated, the cooling power of the tunnel ventilation predicted by the model (blue line in Figure 11) also decreased since the required amount of cool fresh air to control the indoor temperature was reduced. Furthermore, the model prediction indicated that evaporative cooling wss activated again the next day at approximately 1189 h, but it was not recorded by the sensors. One possible reason is that due to the high relative humidity in Chengdu city, the cooling pads, which were wetted at 1168 h, were still effective at 1189 h, providing low temperature air to the poultry house.

**Figure 12:**
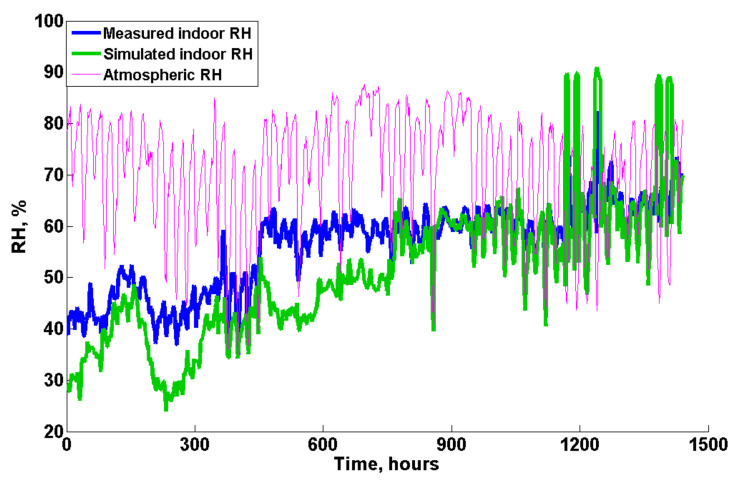
Relative humidity validation results.

In terms of the relative humidity (RH), the atmospheric RH fluctuated between approximately 60 and 80% during the whole validation test, as shown in Figure 12. This high atmospheric RH prevented the wetted cooling pads from quickly drying, which might be responsible for the deviation between the supply air temperature in reality and model, resulting in the difference in the indoor temperature and activation of the evaporative cooling system. Moreover, as shown in Figure 12, the customized model underestimated the indoor RH values at the beginning of the batch (from 0~800 h) compared with the monitored data. One possible reason for the higher monitored indoor RH values is the placement of the bell drinkers in reality at the beginning of the batch, which to some extent would add more moisture to the air through evaporation. Additionally, the moisture in the manure was not considered in the model and might also be responsible for a relatively large proportion of the moisture source at the early stage. Furthermore, it is also hypothesized that the bird’s heat production at the early stage is underestimated by Equation (1), which results in the underestimation of the flock vapor mass production rate calculated from Equation (11). Nevertheless, the simulated indoor RH matched well with the measured data from approximately 800 to 1440 h, showing limited discrepancies. Additionally, it should be noted that when evaporative cooling was activated in the model, the indoor RH would increase to approximately 88%, as shown in Figure 12, at approximately 1200 h and at the end of the test.

The goodness-of-fit of the model for temperature (T) and relative humidity (RH) was statistically evaluated by using the root mean square error (RMSE) and mean absolute error (MAE), as shown in Table 9. The RMSEs of the hourly T and RH were 0.93 °C and 9.41%, respectively. The MAE was calculated to be 0.73 °C for the hourly indoor temperature and 7.36% for RH. The above statistical indices were considered to be satisfactory according to Costantino et al. [24]. Furthermore, with regard to the averaged daily values, all errors were lower than the hourly values, indicating a better fit.

### 4.2. Indoor Gas Concentration

**Figure 13:**
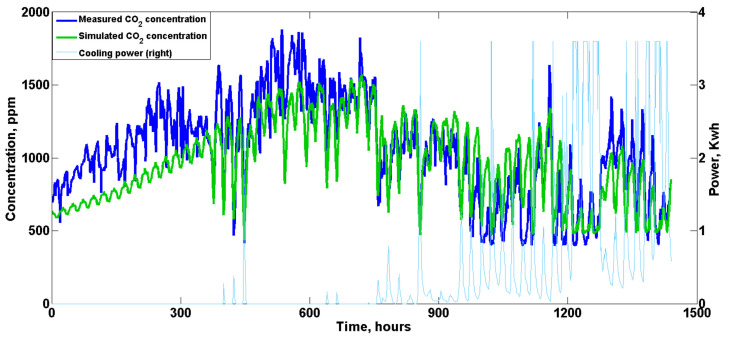
Measured and simulated indoor ${\mathrm{CO}}_{2}$ concentrations during the validation test. Note: The base ventilation is not included in the figure. The maximum rated power of tunnel ventilation was 3.6 KW.

The results of the indoor ${\mathrm{CO}}_{2}$ concentration are shown in Figure 13. Since the priority is to keep the house warm at the start of the batch, only the base ventilation was activated to ensure the minimum ventilation requirement; therefore, both measured and predicted indoor ${\mathrm{CO}}_{2}$ concentrations indicated a gradual increasing trend from the beginning of the test to approximately 750 h due to the increase in bird body weight. The maximum concentration reached approximately 1800 ppm in reality, while the predicted maximum value was approximately 1500 ppm, as shown in Figure 13. The tunnel ventilation was activated for cooling when the birds grew up and were no longer vulnerable to the cold air, and this considerably affected the indoor gas concentration as expected. The fluctuation of daily indoor ${\mathrm{CO}}_{2}$ concentration followed the cooling power (ventilation rate) curve at the middle and end of the validation test, indicating a daily average concentration of approximately 800 ppm. Moreover, we infer that the relatively large discrepancies noted at the beginning of the test are also due to the underestimation of animal heat production predicted by Equation (1). In general, the estimated ${\mathrm{CO}}_{2}$ concentration followed the overall trend recorded by sensors and matched well with the real data as the birds grew up.

**Figure 14:**
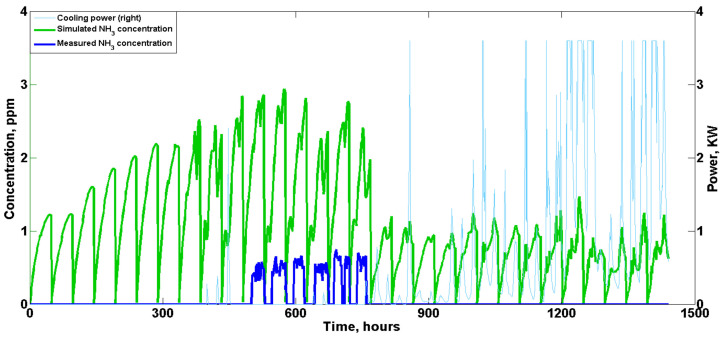
Simulated indoor NH3 concentration during the validation test. Note: The base ventilation is not included in the figure. The maximum rated tunnel ventilation power was 3.6 kW.

In terms of the indoor ${\mathrm{NH}}_{3}$ concentration, notably, ${\mathrm{NH}}_{3}$ was detected by the sensors only for a few days at the middle of the validation test as shown in Figure 14. During about 500~750 h, the measured indoor concentrations were extremely low with an averaged daily value of about 0.5 ppm and no clear overall trend was found. In addition, the ${\mathrm{NH}}_{3}$ concentrations showed a cyclical pattern resulting from the manure removal performed every 48 h, which is similar to that estimated by the model. Although only the base ventilation fan was activated at the beginning of the flock (0~500 h), the birds were small, producing limited manure and ${\mathrm{NH}}_{3}$, which could not be effectively detected by the sensors. Meanwhile, due to the activation of the tunnel ventilation from about 750 to 1440 h, the indoor ${\mathrm{NH}}_{3}$ concentration was also kept at extremely low levels that could hardly be detected by the sensors. Finally, it should be noted that in the present study the sensors were fixed to the ceiling of the house; the dilution of the ${\mathrm{NH}}_{3}$ concentration during the propagation process in reality would make it even harder to be effectively detected as the original concentration was very low. In contrast, the model estimated an overall increased trend of indoor ${\mathrm{NH}}_{3}$ concentrations at the beginning of the test (0~750 h), showing a maximum averaged daily value of approximately 1.5 ppm. With the activation of tunnel ventilation, the predicted daily averaged ${\mathrm{NH}}_{3}$ concentration was lowered to approximately 0.5 ppm, which is far below the threshold value of 25 ppm for poultry houses. Both the measured and simulated results demonstrate very low indoor ${\mathrm{NH}}_{3}$ concentrations during the whole validation test, indicating the current ventilation strategies could efficiently remove the harmful indoor gases.

As shown in Table 10, the corresponding RMSE for hourly ${\mathrm{CO}}_{2}$ and ${\mathrm{NH}}_{3}$ was 226.77 and 1.18 ppm, respectively. When daily values were calculated and statistically evaluated, lower RMSE and MAE values were obtained, indicating a better fit on a daily basis. Overall, considering that human factors, including inspection, cleaning, vaccination, etc., would to some extent affect indoor gas concentrations (air flow caused by personnel movement) and are not included in the purely theoretical customized model, the simulated results are satisfactory, showing similar overall trends and acceptable discrepancies.

### 4.3. Energy Consumption

**Figure 15:**
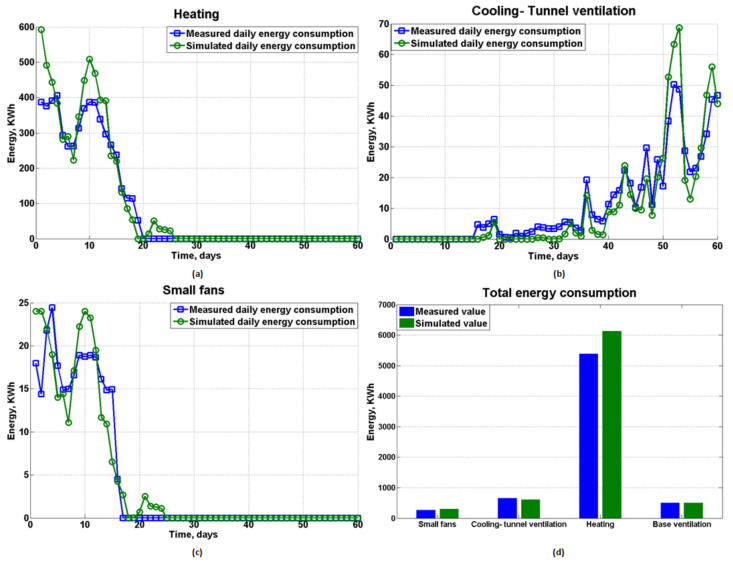
Daily energy consumption and total energy consumption for different aspects. (**a**) Heating; (**b**) Cooling-tunnel ventilation; (**c**) small fans and (**d**) Total energy consumption.

The overall energy consumption due to the environmental control in the poultry house for different aspects is illustrated in Figure 15. As shown in Figure 15a, the predicted daily heating energy matched well with the measured data except during the first few days. The discrepancies noted at the start of the batch are probably due to the underestimation of the animal heating production based on Equation (1), which might to some extent be responsible for the higher heating requirement in the model. Regarding tunnel ventilation and small fans, as shown in Figure 15b,c, the estimated energy consumption showed good agreement with the monitored value both in terms of the overall trend and absolute value. Finally, the total energy consumption for the 60-day validation test is shown in the bar chart in Figure 15d. The corresponding differences between the measured and simulated energy consumption for heating, tunnel ventilation, base ventilation and small fans were 13.7%, 7.5%, 0.1% and 13.3%, respectively. Since the base ventilation was kept activated at the rated power at all times both in reality and in the model, almost no difference was found. The majority of the energy was consumed by heating in reality, accounting for approximately 79.0%, followed by tunnel ventilation (9.7%) and base ventilation (7.4%). In summary, the total energy consumption predicted by the model was 7547.5 kWh, while the measured value was 6824.2 kWh, demonstrating a difference of approximately 10.6%, which is acceptable considering that various human factors were not considered in the theoretical model. Finally, since the solar irradiance in Chengdu city is very limited, with a recorded daily maximum value of approximately $800\;W/m^{2}$ during the validation test, taking the solar radiation into consideration would only have had a 0.6% impact on the total energy demand in the present study.

## 5. Conclusions

A customized model with a time step of one hour was built in the present study to provide an efficient method to estimate the energy consumption of poultry farming under indoor environmental control conditions. The energy balance solution followed the simple hourly method described in ISO 13790, and the indoor gas concentration, including carbon dioxide and ammonia, was also simultaneously determined based on recently developed models or equations. A 60-day continuous measurement campaign was performed in an experimental layer house to validate the proposed model. Dozens of sensors were used in the layer house to collect the indoor and outdoor environmental data, including temperature, relative humidity, solar radiation and gas concentrations. Additionally, several electricity meters were applied in the electrical lines to monitor the energy consumption from ventilation, heating and other components. All the data from sensors and meters were transmitted to a central processing/recording system, and the historical data could be reviewed at any time during/after the test. By comparing the monitored data with that estimated by the customized model, several conclusions can be drawn as follows:

1. The simulated indoor temperature and relative humidity matched well with the monitored data showing similar overall trends. The average RMSEs of the daily T and RH were $0.93\;{}^{\circ}C\;\left(3.3\%\right)$ and 9.41%, respectively, indicating acceptable discrepancies according to a previous study [23].
2. The indoor ${\mathrm{CO}}_{2}$ concentration showed good agreement with the real data, especially for the second half of the validation test when the birds grew up. The tunnel ventilation played a crucial role in affecting the ${\mathrm{CO}}_{2}$ concentration as expected, and the final indoor daily average concentration stabilized at approximately 800 ppm.
3. The indoor ${\mathrm{NH}}_{3}$ concentration showed a clear cyclical pattern resulting from manure removal performed every 48 h. The model predicted the daily averaged concentration was approximately ~1.5 ppm at the beginning of the batch, and the value decreased to approximately 0.5 ppm at the end due to the activation of tunnel ventilation. Ammonia gas was only detected by the sensors for a few days in reality with an averaged daily value of about 0.5 ppm, which is far below the threshold value. Both estimated and measured results of indoor ${\mathrm{NH}}_{3}$ concentrations demonstrated that the current ventilation strategies can effectively and efficiently remove indoor harmful gases.
4. The corresponding difference between the measured and simulated energy consumption for heating, tunnel ventilation and base ventilation was 13.7%, 7.5% and 0.1%, respectively. The difference in total energy consumption was approximately 10.6%, indicating an acceptable discrepancy as suggested by the previous research [24], especially considering that many nonavoidable human interventions occur during the actual production process.

The validation results demonstrate that the customized model could correctly simulate the indoor environment including indoor gas concentrations during poultry farming and accurately predict the total energy consumption with limited discrepancies. The validated model enables producers to quickly optimize their production planning and management strategies and increase the production rate of unit energy consumption, achieving precision livestock farming from an energy consumption perspective.

## 6. Future Study

- Quantify the difference in total energy consumption (and greenhouse gas emissions) among several typical management strategies. Conduct the cost-benefit analysis at the same time to provide the optimum strategy for the producers from a more comprehensive perspective.
- Incorporate the module for an indoor environment early warning function into the model by taking the 48 h weather forecast data into consideration.

## Figures and Tables

**Table 1:** The thermal-physical parameters of the main elements of the poultry house.

Element	$\mathrm{Area}\;(m^{2})$	Material	$\mathrm{Heat}\;\mathrm{Transfer}\;\mathrm{Coefficient}\;(W\;m^{-2}\cdot K^{-1})$
Roof	368.0	Rock-wool insulation board	0.35
Wall	229.2	Firebrick	1.05
Windows	4.6	Polystyrene + metal frame	6.67
Door	5.8	Single-decker wood	4.76
Ground	368.0	Concrete	1.20
Ventilation fans	6.4	/	10.0

**Table 2:** Animal body weight and feeding strategies applied in the present study.

Age (Days)	Age (Weeks)	$\mathrm{Body}\;\mathrm{Weight},\;W_{b}\;(g)$	Feed (g/bird·day)
0	0	/	/
7	1	100	ad libitum feeding
14	2	210	ad libitum feeding
21	3	350	ad libitum feeding
28	4	520	ad libitum feeding
35	5	640	48
42	6	760	50
49	7	860	52
56	8	960	55
63	9	1050	57
70	10	1140	60
77	11	1230	63
84	12	1320	67
91	13	1410	70
98	14	1500	74
105	15	1590	78
112	16	1680	83
119	17	1770	88
126	18	1860	93
133	19	1950	98
140	20	2040	103

**Table 3:** Coefficients for Equation (8) used for calculating the sensible fraction of the heat emission.

Coefficient	Value	Unit
$f_{1}$	$-1.454\times 10^{-7}$	${{}^{\circ}C}^{-5}$
$f_{2}$	$+1.565\times 10^{-5}$	${{}^{\circ}C}^{-4}$
$f_{3}$	$-6.795\times 10^{-4}$	${{}^{\circ}C}^{-3}$
$f_{4}$	$+1.422\times 10^{-2}$	${{}^{\circ}C}^{-2}$
$f_{5}$	$-1.416\times 10^{-1}$	${{}^{\circ}C}^{-1}$
$f_{6}$	+1.234	/

**Table 4:** Coefficients for estimating the manure coverage proportion.

Coefficient	Value
$P_{1}$	$-3.359\times 10^{-5}$
$P_{2}$	$3.621\times 10^{-3}$
$P_{3}$	−0.1648
$P_{4}$	5.081
$P_{5}$	−4.105

**Table 5:** Aspects considered in the model for the final energy consumption calculation.

Aspects	Notes	Unit
Heating	$\mathrm{Consider}\;\mathrm{the}\;\mathrm{coefficient}\;\mathrm{of}\;\mu_{\mathrm{boiler}}$ $\;\mathrm{and}\;\mu_{\mathrm{pipe}}$	kWh
Forced convection heat transfer	Electrical energy consumed by the small fans located at the back of the radiators	kWh
Radiator natural heat convection	During the first few weeks, radiators were treated as heat sources	kWh
Base ventilation	Electrical energy consumed by the base ventilation fan	kWh
Tunnel ventilation	Electrical energy consumed by the three tunnel ventilation fans	kWh
Other (including evaporative cooling system, manure belt cleaning, feeding, etc.)	Neglected	N/A

**Table 6:** Environmental parameters monitored during the validation test.

Parameters	Method	Unit	Information
Indoor air temperature	Temperature sensors (±0.2)	°C	Model SHT20, Huakong Xingye Technology, Beijing, China
Indoor RH	RH sensors (±1)	%	Model SHT20, Huakong Xingye Technology, Beijing, China
$\mathrm{Indoor}\;{\;\mathrm{CO}}_{2}$ concentration	Gas sensors (±5)	ppm	Model 336, Huakong Xingye Technology, Beijing, China
$\mathrm{Indoor}\;{\;\mathrm{NH}}_{3}$ concentration	Gas sensors (±0.5)	ppm	Model 458, Zhize, Jinan, China
Indoor air velocity	Portable speed sensors (±0.1)	m/s	Model 9545, TSI, USA
Outdoor air temperature	Portable meteorological station (±0.2)	°C	Model SHT20, Huakong Xingye Technology, Beijing, China
Outdoor RH	Portable meteorological station (±1)	%	Model SHT20, Huakong Xingye Technology, Beijing, China
Solar radiation intensity	Solar radiometer (±2)	$w/m^{2}$	Model HSTL-ZFSQ, Huakong Xingye Technology, Beijing, China

**Table 7:** Energy consumption monitored in the validation test and applied in the model validation.

Electrical Energy Consumption	Method	Unit	Notes
Tunnel ventilation fans	Meter	kWh	Rated power is 1200 W/fan $,\;\sim 42,000{\;m}^{3}/h$
Base ventilation fan	Meter	kWh	Rated power is 350 W $,\;\sim 6000{\;m}^{3}/h$
Boiler	Meter	kWh	Rated power is 45 kW
Small fans lactated at the back of the radiators	Meter	kWh	Rated power is 100 W/fan

**Table 8:** Other parameters measured in the case study for model validation.

Parameters	Method	Unit	Notes
Animal body weight	Weight scale (±1)	g	To ensure the averaged value follows the designed body weight curve (Table 2)
Manure pH	Quality certified laboratory	/	Measured once a week, model input
Manure MC	Quality certified laboratory	%	Measured once a week, model input

**Table 9:** Statistical indices for evaluating the goodness-of-fit of the model in terms of the temperature (T) and relative humidity (RH).

		RMSE	MAE
Hourly basis	T	0.93 °C	0.73 °C
	RH	9.41%	7.36%
Daily basis	T	0.64 °C	0.51 °C
	RH	8.16%	6.79%

**Table 10:** Statistical indices for evaluating the goodness-of-fit of the model in terms of ${\mathrm{CO}}_{2}$ and ${\mathrm{NH}}_{3}.$

		RMSE	MAE
Hourly basis	${\mathrm{CO}}_{2}$	226.77 ppm	185.86 ppm
	${\mathrm{NH}}_{3}$	1.18 ppm	1.01 ppm
Daily basis	${\mathrm{CO}}_{2}$	190.06 ppm	160.94 ppm
	${\mathrm{NH}}_{3}$	1.15 ppm	1.01 ppm

## Data Availability

Data available on request from the corresponding author.
